# 

**Published:** 1860-02

**Authors:** 


### CONTENTS

OF THE

### Chicago Medical Journal for February, 1860.

### ORIGINAL COMMUNICATIONS.

PAGE.

ARTICLE 1.—Case of Obstructed Labor from an Extososis, and adhesion of Vaginal walls—Operation for cutting the stricture—Craniotomy and delivery—Death on the fifth day. Reported by G. W. Phillips, M. D. - - - - - - - - - - - - - 57
ARTICLE 2.—Cases of Disease of the lower jaw, for which resection and removal of the Parotid and Submaxillary Gland was performed. By D. Brainard, M. D. [Illustrated.] 61
ARTICLE 3.—Diphtheria. By W. G. Dyas, M. D., - - - - - - - - - - 68
ARTICLE 4.—Physiology of the Circulation, - - - - - - - - - - 76
Excerpta from the Medical Press By J. A. A., - - - - - - - - - - - - 80
Chicago Academy of Medical Sciences, - - - - - - - - - - - - - 92
Vesico-Vaginal Fistula—A new operation proposed, by which the twisted suture may be applied By Paul F. Eve, M. D. - - - - - - - - - - - - - - - 102

### SELECTIONS.

Severe Hæmorrhage from the Tonsil, arrested by the solution of perchloride of iron, - - 78
Epistaxis of alarming character arrested by injections of perchloride of iron, by Dr. Fountain 78
Fees for Professional services, - - - - - - - - - - - - - - 95
Bright's Disease of the Kidney, - - - - - - - - - - - - - - - 105
The use of Chloroform and Aconite in Neuralgia, - - - - - - - - - 107
Inversion of the Uterus successfully reduced, - - - - - - - - - - 109
Glycerine Ointment for Itch, - - - - - - - - - - - - - 109
Administration of Medicine to children, - - - - - - - - - - 110
Induction of Premature Labor by Uterine Catheterism, - - - - - - - 111
Iodide of Potassium as an Antiglactic, - - - - - - - - - - 112

### EDITORIAL.

Mystical Medical Politics, - - - - - - - - - 113
Book and Pamphlet Notices, - - - - - - - - 119

### SURGICAL NOTES.

1. Amputation at the Shoulder Joint, - - - - - - - - 114
2. Amputation of Thigh for disease of the Knee Joint, - - - - - 116
3. On the use of Subcataneous Perforation of Bone in Anchylosis of the Knee Joint, - 117

